# Reduction of glutamatergic activity through cholinergic dysfunction in the hippocampus of hippocampal cholinergic neurostimulating peptide precursor protein knockout mice

**DOI:** 10.1038/s41598-022-23846-x

**Published:** 2022-11-10

**Authors:** Kengo Suzuki, Yoshiaki Ohi, Toyohiro Sato, Yo Tsuda, Yuta Madokoro, Masayuki Mizuno, Kenichi Adachi, Yuto Uchida, Akira Haji, Kosei Ojika, Noriyuki Matsukawa

**Affiliations:** 1 Department of Neurology, Graduate School of Medical Sciences, Nagoya City University, 1 Kawasumi, Mizuho-Ku, Nagoya, 467-8602 Japan; 2 Laboratory of Neuropharmacology, School of Pharmacy, Aichi Gakuin University, 1-100 Kusumoto, Chikusa-Ku, Nagoya, 464-8650 Japan

**Keywords:** Neurotrophic factors, Dementia, Ageing

## Abstract

Cholinergic activation can enhance glutamatergic activity in the hippocampus under pathologic conditions, such as Alzheimer’s disease. The aim of the present study was to elucidate the relationship between glutamatergic neural functional decline and cholinergic neural dysfunction in the hippocampus. We report the importance of hippocampal cholinergic neurostimulating peptide (HCNP) in inducing acetylcholine synthesis in the medial septal nucleus. Here, we demonstrate that HCNP-precursor protein (pp) knockout (KO) mice electrophysiologically presented with glutamatergic dysfunction in the hippocampus with age. The impairment of cholinergic function via a decrease in vesicular acetylcholine transporter in the pre-synapse with reactive upregulation of the muscarinic M1 receptor may be partly involved in glutamatergic dysfunction in the hippocampus of HCNP-pp KO mice. The results, in combination with our previous reports that show the reduction of hippocampal theta power through a decrease of a region-specific choline acetyltransferase in the stratum oriens of CA1 and the decrease of acetylcholine concentration in the hippocampus, may indicate the defined cholinergic dysfunction in HCNP-pp KO mice. This may also support that HCNP-pp KO mice are appropriate genetic models for cholinergic functional impairment in septo-hippocampal interactions. Therefore, according to the cholinergic hypothesis, the model mice might are potential partial pathological animal models for Alzheimer’s disease.

## Introduction

In Alzheimer’s disease, episodic memory disturbance is the main clinical symptom^1^. Hippocampal function is crucial for episodic memory function, which is dynamically regulated by the glutamatergic network from the superficial layer (2–3) of the entorhinal cortex to the CA3 tri-synaptic pathway or the CA1 mono-synaptic pathway, then finally to the conclusive hippocampal circuit as efferent fibers from the CA1 to the cortex through the deeper entorhinal cortex (layer 5)^2–4^. Hippocampal glutamatergic activity may be modified by any afferent neural fiber connected to the hippocampus, such as cholinergic networks from the medial septal nucleus (MSN) to the hippocampus.

The local network in the MSN, which is established by cholinergic, glutamatergic, and GABAergic neurons, may project to the glutamatergic pyramidal neurons and different subsets of the GABAergic neurons in the CA1 region of the hippocampus and generate correlated rhythmic activity between 4 and 12 Hz, which is known as theta oscillation and are local field potential fluctuations in the hippocampus^3,5^. Most septal projections from the MSN to the hippocampus originate from cholinergic neurons^6^. The release of acetylcholine from the neural terminals in CA1 can lead to the facilitation of long-term potentiation (LTP) ^7^. Under insufficient activity of the glutamatergic neural network in the hippocampus, the cholinergic neural network from the MSN to the hippocampus may amplify hippocampal glutamatergic neural activity^8–10^. Cholinergic neural dysfunction from the MSN to the hippocampus is confirmed in a variety of neurogenerative disorders, including Alzheimer’s disease and Lewy body disease. The effect of cholinesterase inhibitors in clinics, such as donepezil, rivastigmine, and galantamine hydrobromide, also suggests that the cholinergic network from the MSN to the hippocampus may be crucial for the maintenance of glutamatergic neural activity^11–13^. However, the molecular mechanisms underlying cholinergic activation from the MSN to the hippocampus remain unclear. Moreover, an adequate genetic model for cholinergic functional impairment in septo-hippocampal interactions, for Alzheimer’s research, is unavailable.

Previously, the neural functioning peptide, hippocampal cholinergic neurostimulating peptide (HCNP)^9,14^, was purified from a juvenile rat hippocampus, which induces acetylcholine synthesis via increasing the amount of choline acetyltransferase (ChAT) in the MSN. According to complementary deoxyribonucleic acid (DNA) cloning, this peptide is expected to be aligned at the N-terminal region of the 21 kDa HCNP precursor protein (HCNP-pp), which is composed of 186 amino acids and is also known as Raf kinase inhibitory protein and phosphatidylethanolamine binding protein-1^14–16^. Based on information from the chromosomal DNA, conditional knockout (KO) mice were generated using the Cre-loxP system^17^. When HCNP-pp was decreased by mating the mice with Cre-recombinase transgenic mice driven by the CaMKII promotor, the reduction of acetylcholine (ACh) was confirmed in the hippocampus by microdialysis with a decrease in the vesicular acetylcholine transporter (VAchT)^18^. Theta oscillation may be also reduced in the hippocampus of HCNP-pp KO mice with a decrease in the ChAT positive axonal volume in the stratum oriens (SO) of CA1, which is consistent with cholinergic dysfunction in the MSN as the theta oscillation generator^17^. Also, it has been reported that overexpression of HCNP-pp in the hippocampus may enhance glutamatergic neuronal activity via the muscarinic M1 receptor, which was measured by the slope of the field excitatory postsynaptic potentials (fEPSPs) during LTP, indicating that it is a factor that regulates the hippocampal glutamatergic neurons^19^.

When devising an Alzheimer’s disease model after considering cholinergic dysfunction based on the cholinergic hypothesis^20^, whether glutamatergic neural suppression is related to cholinergic neural dysfunction should be confirmed in the model . Therefore, we hypothesized that fEPSP during LTP in the hippocampus of HCNP-pp KO mice may be decreased with any molecular alteration related to cholinergic function in the MSN-hippocampus network.

In the present study, to verify the appropriateness of HCNP-pp KO mice as partial pathological models for Alzheimer’s disease, we investigated whether HCNP-pp KO mice can inhibiti fEPSP during LTP in the hippocampus. To confirm the involvement of cholinergic dysfunction in LTP inhibition, we also screened any alterations in molecules related to LTP using Western blots.

## Results

### Reduction in the field excitatory postsynaptic potentials in the hippocampus of HCNP-pp knockout via cholinergic dysfunction

In wild mice, cholinergic neuronal stimulation was reported to enhance the slope of fEPSPs in the hippocampal LTP with repeated tetanus bursts (100 Hz, 1 s) on the Schaffer collateral-commissural fibers (SCs) as preconditioning. For the experimental setup conditions, more than double tetanic stimulation (D-TS) with two-second intervals could fully induce the enhancement of the fEPSPs, while the slope of the fEPSP after single tetanic stimulation (S-TS) was insufficiently enhanced. Furthermore, cholinergic activation via the muscarinic M1 receptors played a crucial role in the enhancement of the fEPSP from S-TS and D-TS in wild mice. Based on this, to evaluate the cholinergic function for glutamatergic activity inducing LTP, we used S-TS or D-TS on the SCs as the preconditioning method in this study, following previous studies^10,19^.

**Figure 1:**
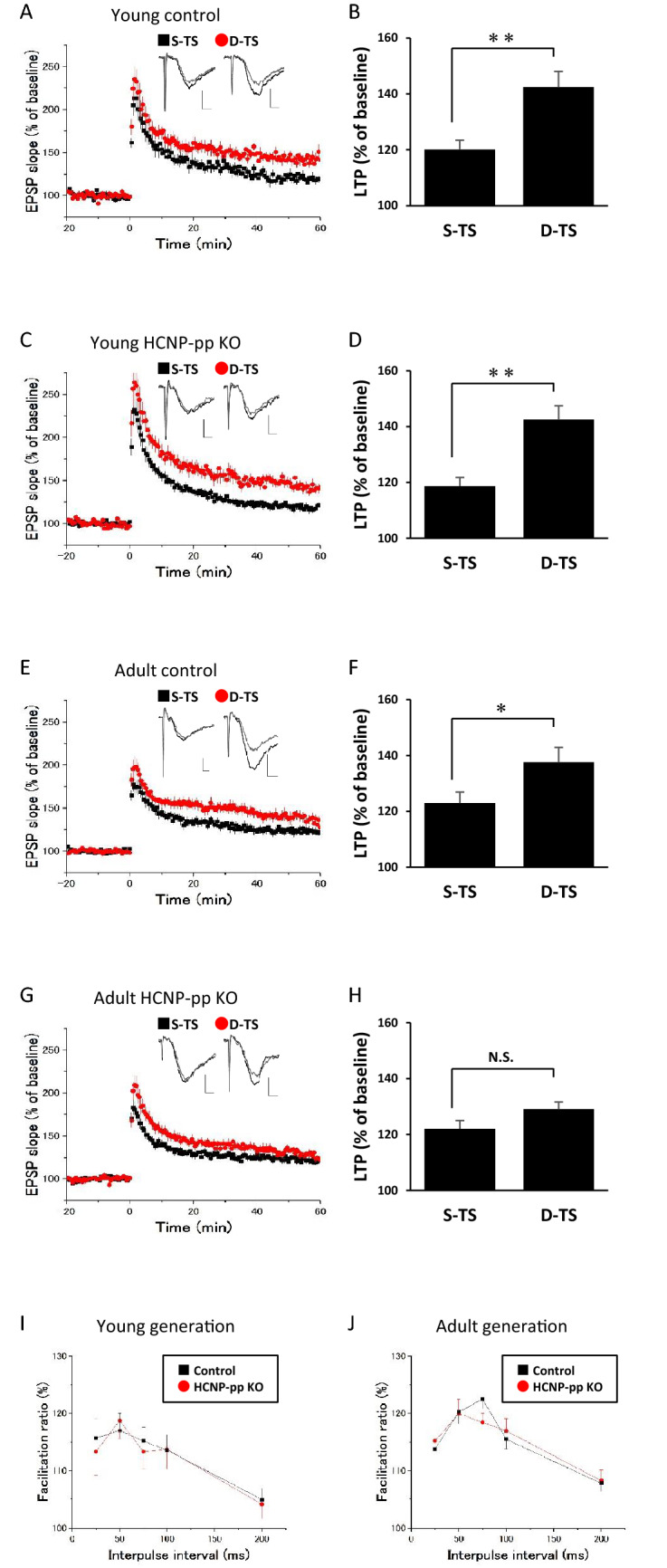
The enhanced effect from single-tetanic stimulation (S-TS) and double-tetanic stimulation (D-TS) in the hippocampus of young and adult hippocampal cholinergic neurostimulating peptide-precursor protein (HCNP-pp) knockout (KO) mice when compared to the age-matched control mice. (**A**, **C**, **E**, and **G**) The time courses of the field excitatory postsynaptic potentials (fEPSPs). Tetanic stimulation (TS; 0.1 ms, 100 Hz for 1 s) was applied to the Shaffer collateral-commissural fibers (SCs) at time 0 with the repetitions indicated (black square; S-TS; red circle; D-TS). Representative traces of the fEPSP were taken from 0 to 10 min before TS (gray line) or 50 to 60 min after TS (black line). (**B**, **D**, **F**, and **H**)(Scale bar; 0.4 mV and 2.5mS) The percentage change in long-term potentiation (LTP) was taken 50–60 min after TS. HCNP-pp KO (young; S-TS n = 14, N = 5, D-TS n = 12, N = 5, adult; S-TS; n = 13, N = 5, D-TS n = 14, N = 9), Control (young; S-TS n = 8, N = 5, D-TS n = 9, N = 5, adult; S-TS n = 15, N = 7, D-TS n = 14, N = 5) (S-TS; black square, D-TS; red circle). (**I** and **J**) The ratio of paired-pulse facilitation in the young or adult generation. Paired-pulse facilitation was measured by applying two closely paired stimuli (interpulse intervals: 25–200 ms), and the ratio of the second response to the first response was plotted against the interpulse interval. The groups include the HCNP-pp KO (red circle; young n = 14, N = 5; adult n = 38, N = 16) and control mice (black square; young n = 24, N = 5; adult n = 41, N = 14). The data are expressed as the mean ± standard error of the mean. *p < 0.05. **p < 0.01.

As a first step, to confirm the similar enhancement effect of the fEPSP from the S-TS and D-TS in the hippocampus of HCNP-pp KO mice, we performed LTP induction using S-TS and D-TS preconditioning in HCNP-pp KO mice at 11–17 weeks old (young generation) in comparison with the age-matched control mice. The enhancement effect from S-TS and D-TS in LTP potentiation was equally shown in the HCNP-pp KO mice when compared to the age-matched control mice (Fig. 1A–D). Based on this data, to assess age-related alteration for an enhancement effect, the same experiment was conducted in HCNP-pp KO mice at 24–35 weeks old (adult generation). The adult generation of HCNP-pp KO mice revealed an impairment in the enhancement effect from S-TS and D-TS when a comparison was made with the age-matched control mice (Fig. 1E–H). These results suggest that the impairment of the enhancement effect from S-TS and D-TS in fEPSP potentiation may be phenotypically manifested as an age-related change from 24 to 35 weeks old in the hippocampus of HCNP-pp KO mice when evaluated against the aged-matched control mice.

Paired-pulse stimulation of the SCs was performed to evaluate alteration in the transmitter secretion efficiency as a presynaptic function. No difference in the facilitation ratio was observed between the HCNP-pp KO and control mice in both the young and adult generation, suggesting that there was no significant age-related alteration in the pre-synaptic glutamatergic transmitter release function in the hippocampus of HCNP-pp KO mice when contrasted with the age-matched control mice (Fig. 1I,J).

**Figure 2:**
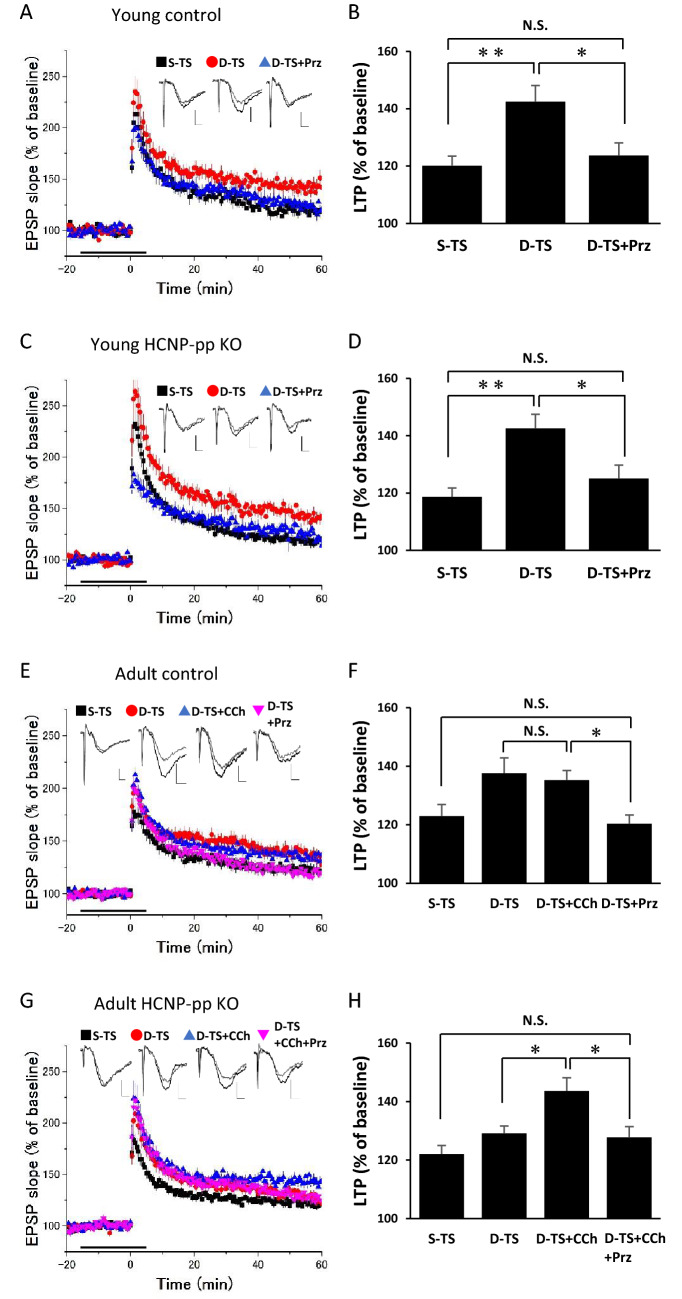
The involvement of cholinergic dysfunction via the M1 receptor signal in the impairment of the enhancement effect from single-tetanic stimulation (S-TS) and double-tetanic stimulation (D-TS) in the hippocampus of adult hippocampal cholinergic neuro stimulating peptide-precursor protein (HCNP-pp) knockout (KO) mice. (**A**, **C**, **E**, and **G**) The time courses of the fEPSP of S-TS, D-TS, and the pharmacological conditions. The stimulation and recording conditions in the experiments were the same as in Fig. 1, and the representative traces of the fEPSP (gray line, 0–10 min before tetanic stimulation [TS]; black line, 50–60 min after TS) and bars are indicated. (**B**, **D**, **F**, and **H**) The percentage change in long-term potentiation (LTP) was taken 50–60 min after TS. (**A**–**D**) The enhancement effect from S-TS and D-TS in the hippocampus of young HCNP-pp KO mice and age-matched control mice with prior application of 0.1 µM Prz (D-TS with Prz; HCNP-pp KO n = 12, N = 4, control n = 9, N = 4) (S-TS; black square, D-TS; red circle, D-TS with Prz; blue triangle). (**E** and **F**) The enhancement effect from S-TS and D-TS in the hippocampus of adult age-matched control mice with prior application of 0.1 µM Prz or 50 nM CCh (D-TS with CCh; n = 15, N = 6, D-TS with Prz; n = 12, N = 6) (S-TS; black square, D-TS; red circle, D-TS with CCh; blue triangle, D-TS with Prz; pink triangle). (**G** and **H**) The enhancement effect from S-TS and D-TS in the hippocampus of adult HCNP-pp KO mice with prior application of 0.1 µM Prz and/or 50 nM CCh (D-TS with CCh; n = 15, N = 8, D-TS with CCh and Prz; n = 14, N = 6) (S-TS; black square, D-TS; red circle, D-TS with CCh; blue triangle, D-TS with CCh and Prz; pink inverse triangle). The data are expressed as mean ± standard error of the mean. *p < 0.05. **p < 0.01.

Next, it was determined whether cholinergic dysfunction might be involved in the impairment of fEPSP enhancement from S-TS and D-TS in the HCNP-pp KO mice. Pharmacological experiments were conducted using carbachol (CCh) (50 nM; a cholinergic agonist) and pirenzepine (Prz) (0.1 µM; a selective muscarinic M1 receptor antagonist), following previous studies^10,19^. The lower amplitude of the fEPSP of D-TS in the hippocampus of the adult HCNP-pp KO mice was enhanced by 50 nM CCh (Fig. 2G,H), whereas no enhancement was observed by 50 nM CCh in the hippocampus of the adult control mice (Fig. 2E,F). Additionally, 0.1 µM Prz could suppress the fEPSP level of D-TS to a similar level as S-TS in the young HCNP-pp KO mice and young and adult control mice (Fig. 2A–F; *p* < 0.05). Then, 0.1 µM Prz was also found to suppress the fEPSP of D-TS with 50 nM CCh to a similar level as S-TS in the adult HCNP-pp KO mice (Fig. 2G,H; *p* < 0.05). This suggests the involvement of cholinergic neuromodulatory dysfunction via the M1 receptor in the impairment of the enhancement effect from S-TS and D-TS in the hippocampus of the adult HCNP-pp KO mice.

### The involvement of vesicular acetylcholine transporter reduction in the pre-synapse in the old generation HCNP-pp knockout mice

**Figure 3:**
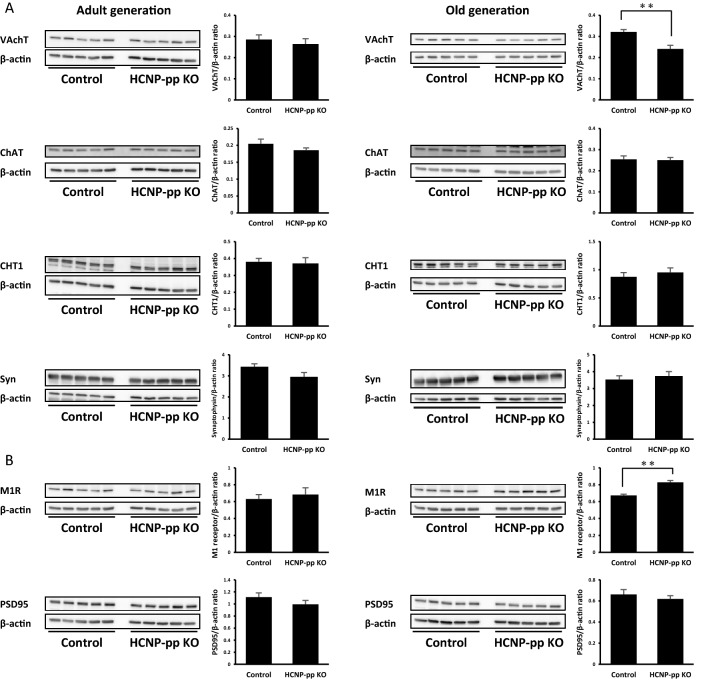
The evaluation of molecules related to cholinergic synaptic function screened using western blots. (**A**) Choline acetyltransferase (ChAT), vesicular acetylcholine transporter (VAchT), high-affinity choline transporter (CHT1), synaptophysin, (**B**) muscarinic M1 receptor evaluated as molecules related to cholinergic synaptic function. (**A** and **B** left panel) The adult generation of hippocampal cholinergic neurostimulating peptide-precursor protein knockout (HCNP-pp KO) mice and age-matched control mice. (**A** and **B** right panel) The old generation HCNP-pp KO mice compared to the age-matched control mice (*p* < 0.01). Five HCNP-pp KO mice and five age-matched control mice were examined in each generation. The β-actin as internal standard in each blot was subsequently assessed on the same sheet (Fig. S2). The data are presented as the mean ± standard error of the mean. *p < 0.05. **p < 0.01.

Next, we screened the involvement of some of the molecules that are associated with the cholinergic and/or glutamatergic terminals in cholinergic neuromodulatory dysfunction in the hippocampus of adult HCNP-pp KO mice using Western blots. Despite our prediction, no significant change was shown in the adult HCNP-pp KO mice for all the compounds that were screened in this study (Fig. 3A,B left panel, Fig. S1 left panel). Under the expectation of a time-dependent change in a similar fashion to the electrophysiological experiments, the same screening was investigated in HCNP-pp KO mice at 54–55 weeks old (old generation). The Western blots revealed a significant reduction in VAchT in the old generation HCNP-pp KO mice when measured against that of the age-matched control mice; however, this was not observed for ChAT, high-affinity choline transporter (CHT1), or synaptophysin (Fig. 3A right panel; *p* < 0.05). Interestingly, the amount of muscarinic M1 receptor was significantly increased in the old generation HCNP-pp KO mice when compared to the age-matched control mice (Fig. 3B right panel; *p* < 0.05). In terms of the glutamatergic post-synaptic terminals, no significant change was shown in both the N-Methyl-D-aspartic acid receptor (NMDAR) and α-3-hydroxy-5-methyl-4-isoxasole propionic acid receptor (AMPAR) in the old generation HCNP-pp KO mice when contrasted with the age-matched control mice (Fig. S1 right panel).

## Discussion

In this study, we demonstrated 1) the impairment of the enhancement effect from S-TS and D-TS in fEPSP potentiation during the adult period of 24–35 weeks old (but not during the young period of 11–17 weeks old) was induced in the hippocampus of HCNP-pp KO mice when evaluated against that in the age-matched control mice. 2) A significant reduction in VAchT with a muscarinic M1 receptor increase was revealed in the old generation HCNP-pp KO mice at 54–55 weeks old (but not in the adult HCNP-pp mice) when a comparison was made with the age-matched control mice. 3) A similar presynaptic glutamatergic transmitter release function was electrophysiologically shown in the HCNP-pp KO mice in both the young and adult generations in contrast with that in the control mice. In the Western blots, the typical molecules-related glutamatergic functions were also not changed in the hippocampus of the HCNP-pp KO mice when contrasted with the age-matched control mice in both the adult and old generations.

The functional peptide, HCNP, was originally purified from the soluble fraction of the rat hippocampus and induces acetylcholine synthesis via increasing the amount of ChAT in the MSN^9^. It is cleaved from 21 kDa HCNP-pp and is composed of 186 amino acids^14^. The concentration of ACh in the hippocampus of the HCNP-pp KO mice was reduced with decreasing VAchT^18^. Furthermore, theta oscillation may be also reduced in the hippocampus of HCNP-pp KO mice with a regiospecific decrease in the ChAT positive axonal volume in the stratum oriens of CA1^17^. Therefore, our previous data may indicate that HCNP-pp KO mice are an adequate genetic model for cholinergic functional impairment in septo-hippocampal interactions.

In this study, we confirmed that the amplifying effect of the fEPSP from S-TS and D-TS may be decreased in the hippocampus of adult HCNP-pp KO mice when measured against the age-matched control mice. The impairment of the amplifying effect of the fEPSP from S-TS and D-TS may be pharmacologically involved in the muscarinic receptor, M1. These results agree with a previous study; the overexpression of HCNP-pp in the hippocampus enhances the fEPSPs via the muscarinic M1 receptor through an opposite effect^19^. A western blot analysis determined that postsynaptic glutamatergic modulation may not be involved in the impairment of the amplifying effect of the fEPSP from S-TS and D-TS in this current model. Moreover, presynaptic glutamatergic plasticity may not participate in this phenomenon, which was determined by an electrophysiological experiment, with paired-pulse stimulation of the SCs. The data in this study suggest that the impairment of the amplifying effect of the fEPSP from S-TS and D-TS in the hippocampus of the HCNP-pp KO mice may result from cholinergic plasticity itself, which is consistent with previous studies^17–19^.

In this study, we also confirmed a time-dependent change in both the electrophysiological and biochemical experiments, even though electrophysiological impairment occurred at an earlier time phase than molecule alteration in the hippocampus of the HCNP-pp KO mice when compared to that in the age-matched control mice. Both an age-related cholinergic decline and GABAergic increase in the cell number were revealed in the MSN of rats at 22 months old, while the expression of the cholinergic receptor, α7 nicotine receptor, M1 receptor, GABA-a receptor, glutamatergic receptors, NMDAR subunit 2B (NR2B), and GluR1 in the hippocampus of rats was decreased with age^21,22^. A binding assay found that VAchT may also decrease in the hippocampus with age^23^. The strengthening mechanism of the existing synapse, which is correlated with memory in the hippocampus, might change with age^24^. Some modified mice models, which are related to neurotrophic factors, nerve growth factor (NGF), tropomyosin receptor kinase A, p75 neurotrophin receptor, and Brain-derived neurotrophic factor, may also display age-related change in the electrophysiological/behavioral phenomenon and/or functional molecules associated with neuronal plasticity^25–30^. In this study, the results of electrophysiological and biochemical alteration with age were consistent with those of previous studies on vital age-related change.

Our mice models, HCNP-pp KO mice, electrophysiologically revealed the impairment of cholinergic plasticity from the MSN to the hippocampus with the reduction of the ACh concentration. The regiospecific decrease in the ChAT positive volume in the neuronal terminals in the SO of CA1 was reported as a molecular mechanism underlying cholinergic impairment in this mouse model^17,18^. The amount of VAchT may be declined in the hippocampus of old generation HCNP-pp KO mice in contrast with the age-matched control mice, which is a similar finding to a previous study^18^. Interestingly, we confirmed an increase in the amount of M1 receptor with VAchT decline in the old generation HCNP-pp KO mice when evaluated against the age-matched control mice. The deficiency in the cholinergic innervation from the MSN to the hippocampus can upregulate the amount of M1 receptors in other models, such as the fimbria-fornix lesioned model and basal forebrain cholinergic lesion model with 192IgG-saporin^31,32^. The deprivation of NGF and the expected induction of cholinergic dysfunction in the hippocampus can also increase the density of the M1 receptor in the hippocampus^33^. In contrast, a chronic forced increase in the ACh concentration in the synaptic cleft by the acetylcholinesterase inhibitor or acetylcholinesterase knockout may down-regulate the M1 receptor amount^34,35^. Whereas the depletion of the neuronal transmitter, ACh, might upregulate the sensitivity of the post-synapse due to an increase in the M1 receptor as compensatory regulation in the hippocampus of the HCNP-pp KO mice, lesion model of cholinergic innervation, or NGF depletion model.

The primary limitation of this study is that we could not directly exclude the involvement of glutamatergic or GABAergic activation in the impairment of the amplifying effect of the fEPSP from S-TS and D-TS in the hippocampus of the adult HCNP-pp KO mice. The mechanism of VAchT decline could also not be demonstrated in the old generation HCNP-pp KO mice. In the future, further experiments are needed to elucidate the function of HCNP and/or HCNP-pp in the synaptic plasticity of the hippocampus, including cholinergic presynaptic function.

In conclusion, the present results suggest that the reduction of the ACh concentration in the hippocampus of the HCNP-pp KO mice suppressively modulates postsynaptic muscarinic modulation of LTP enhancement, although the amount of M1 receptor is increased as a compensatory regulation. In combination with previous studies, the results may support that HCNP-pp KO mice are an adequate genetic model for cholinergic functional impairment in septo-hippocampal interactions. Thus, the model mice could also be utilized as partial pathological animal models for Alzheimer’s disease.

## Materials and methods

All the experiments were performed in accordance with the ARRIVE guidelines.

### Animals

The animal experiments were approved by the Animal Care and Use Committee of Nagoya City University Graduate School of Medical Sciences (permit number 18149, 19-017H02) and conformed to the guidelines for the use of laboratory animals which was published by the Japanese government (Law No. 105, October 1973). The generation of homozygous HCNP-pp KO mice and littermate HCNP-pp floxed control mice was performed as previously reported^36^. The animals were housed in specific pathogen-free conditions with a 12 h light/dark cycle (with the lights on from 08:00 to 20:00) and were given free access to food and water.

We used 176 slices from 80 male mice for the electrophysiological experiments for LTP (young generation, 11–17 weeks old; control, n = 17, N = 10, HCNP-pp KO, n = 26, N = 10; adult generation, 24–35 weeks old; control, n = 65, N = 28, HCNP-pp KO, n = 68, N = 32), and 20 female mice were used for Western blot analysis (adult generation, 24–27 weeks old, control, N = 5, HCNP-pp KO, N = 5; old generation, 54–55 weeks old, control, N = 5, HCNP-pp KO, N = 5). Subsequently, paired-pulse facilitation was studied using selected sample slices used in the electrophysiological experiments for LTP (young generation, control n = 24, N = 5, HCNP-pp KO, n = 14, N = 5; adult generation, control, n = 41, N = 14, HCNP-pp KO, n = 38, N = 16).

### Antibodies

The rabbit polyclonal anti-mouse/rat HCNP (HCNP-pp) antibody was generated as previously described^14^. Then, the anti-HCNP-pp antibody was purified with an HCNP affinity column prepared with a Hitrap NHS-activated HP Column (GE Healthcare, Waukesha, WI, USA). The following antibodies were obtained commercially: rabbit monoclonal anti-human/mouse synaptophysin antibody (Abcam, Cambridge, UK), mouse monoclonal anti-rat/mouse postsynaptic density protein 95 (PSD95) antibody (Merck-Millipore, Billerica, MA, USA), goat polyclonal anti-human/mouse ChAT antibody (Merk-Millipore), rabbit polyclonal anti-mouse VAchT/SLC18A3 antibody (NOVUS biological, Littleton, CO, USA), rabbit polyclonal anti-human/mouse CHT-1 antibody (Merck-Millipore), mouse monoclonal anti-β-actin antibody (Sigma, St. Louis, MO, USA), rabbit polyclonal anti-human/mouse muscarinic receptor 1 antibody (Sigma), rabbit polyclonal anti-rat/mouse NR-1 antibody (Sigma), rabbit polyclonal anti-rat/mouse NR-2A antibody (Merck-Millipore), rabbit polyclonal anti-mouse NR-2B antibody (Merck-Millipore), rabbit polyclonal anti-human/mouse GluA1 antibody (Merck-Millipore), and rabbit polyclonal anti-rat/mouse GluA2/3 antibody (Merck-Millipore) for the Western blots, and HRP-conjugated anti-rabbit (MP Biomedicals, Santa Ana, CA, USA), anti-mouse (MP Biomedicals), or anti-goat (Abcam) IgG antibodies were used as secondary antibodies.

### Slice preparation

Slice preparation was performed following a previous study^10,19^. Briefly, the mice were deeply anesthetized with halothane and decapitated. The brains were quickly removed and transverse hippocampal slices with a 400 µm thickness were prepared using a vibrating slice cutter (Linear Slicer Pro 7, Dosaka, Kyoto, Japan) in an ice-cold solution containing (mM): sucrose, 260; KCl, 3; NaH_2_PO_4_, 1.25; NaHCO_3_, 26; D-glucose, 10; and MgCl_2_, 1 (pH 7.4), which was continuously bubbled with 95% O_2_/5% CO_2_. One to four slices were obtained from each animal and one experiment was performed on each slice. The hippocampal slices were incubated for 30 min in artificial cerebrospinal fluid (ACSF) containing (mM): NaCl, 125; KCl, 2.5; CaCl_2_, 2.4; MgCl_2_, 1; NaH_2_PO_4_, 1.25; NaHCO_3_, 25; and D-glucose, 12.5, which was saturated with 95% O_2_/5% CO_2_ (pH adjusted to 7.4) at 32 °C. The slices were kept at room temperature (25 ± 1 °C) in the ACSF at least for 1 h until they were ready for recording.

### Electrophysiology

The electrophysiological study was conducted as per previously published studies^10,19^. Briefly, the slices were fixed in a recording chamber (~ 0.4 mL volume, RC-26GLP, Warner Instruments, Hamden, CT, USA) under a nylon mesh attached to a stainless-steel anchor, and then they were submerged in and continuously perfused with ACSF at a flow rate of 2 ml/min. All the experiments were performed at room temperature (25 ± 1 °C). The fEPSPs were recorded from the stratum radiatum (SR) in the CA1 field using borosilicate glass electrodes (3–5 MΩ) filled with perfusing ACSF. The recordings were made using an Axopatch 200B amplifier (Axon Instruments, Foster City, CA, USA) with a high-cut filter at 2 kHz.

A stainless steel concentric bipolar electrode (Unique Medical, Tokyo, Japan) was placed on the SR, and the SCs were stimulated with a 0.1 ms pulse every 30 s. At the beginning of each experiment, a stimulus–response curve was established, and the stimulus intensity was adjusted to evoke 30–50% of the maximal response, which fell within the stimulus intensity of 20–40 µA. Long-term potentiation in the CA1 region was induced from S-TS or D-TS (0.1 ms pulse duration, 100 Hz for 1 s) of the SCs. The tetanus stimulation in D-TS was performed with two-second intervals. The fEPSPs were recorded in CA1 for 60 min after the induction of LTP. Then, paired-pulse facilitation was studied by applying a pair of stimulations at varying interpulse intervals (25–200 ms).

### Drug application

The drugs Prz and CCh were commercially purchased (Sigma). All the drugs were dissolved in ACSF and applied by gravity-fed 60 mL reservoirs bubbled with 95% O_2_–5% CO_2_ for 10 min just before the induction of LTP.

### Western blot analysis

The Western blot was performed following a previous study^17^. In brief, under deep pentobarbital anesthesia, each mouse was transcardially perfused with phosphate-buffered saline. After the brains were removed and placed on ice, the hippocampi were dissected and immediately frozen in liquid nitrogen and stored at −80 °C until further use. The frozen hippocampi from five HCNP-pp KO mice and five control mice were homogenized in four volumes of lysis buffer containing 30 mM Tris–HCl (pH 8.5), 7 M urea, 2 M thiourea, 4% w/v CHAPS, and a protease inhibitor cocktail (Roche Applied Science, Indianapolis, IND, USA). After incubation for 60 min on ice, the homogenates were centrifuged at 15,000 × *g* for 3 min at 4 °C. After the protein content was measured using the Bradford assay (Pierce, Rockford, IL, USA), the supernatants were stored at −80 °C until further use. Then, 10 μg of each supernatant fraction was loaded onto each lane of 10% gel for SDS–polyacrylamide gel electrophoresis. After electrophoresis, the samples were transferred to Hybond-P membranes (GE Healthcare, Tokyo, Japan) using 25 mM Tris, 192 mM glycine, 0.1% SDS, and 10% methanol as a transfer buffer. Then, the membranes were incubated with 1:500 goat polyclonal anti-ChAT antibody, 1:5,000 rabbit polyclonal anti-VAchT antibody, 1:1,000 rabbit polyclonal anti-CHT-1 antibody, 1:200,000 rabbit polyclonal anti-synaptophysin antibody, 1:2,000 rabbit polyclonal anti-NR-1 antibody, 1:5,000 rabbit polyclonal anti-NR-2A antibody, 1:1,000 rabbit polyclonal anti-NR-2B antibody, 1:5,000 rabbit polyclonal anti-GluA1 antibody, 1:1,000 rabbit polyclonal anti-GluA2/3 antibody, 1:2,000 mouse monoclonal anti-PSD95 antibody, 1:2,000 rabbit polyclonal anti-muscarinic receptor 1 antibody, or 1:50,000 mouse monoclonal anti-β-actin antibody. The membranes were then probed with horseradish peroxidase-conjugated anti-rabbit, anti-mouse, or anti-goat IgG antibodies. The immunoreactive bands were visualized using the ECL Advance Western Blotting Detection kit (GE Healthcare, Tokyo, Japan) and recorded using ImageQuant LAS 4000 (GE Healthcare, Tokyo, Japan). The Western blots were quantified using Amersham Imager 600 Analysis Software (GE Healthcare, Tokyo, Japan).

### Data acquisition and statistical analysis

The data sampling and statistical analysis were performed following previous studies ^10,19^. In brief, all the data were included in the final analyses except for when the baseline response changed during the experiment. The fEPSPs were sampled online at 4 kHz (PowerLab, AD Instruments, Sydney, Australia), stored on a hard disk, and analyzed offline with Scope 4 and Chart 5 (AD Instruments). The maximal slope values of the initial rising phase of the fEPSPs were measured to avoid the contamination of the voltage-dependent components as much as possible. The magnitude of LTP was calculated as a percentage of the averaged fEPSP slope values from 50 to 60 min after each tetanic stimulation relative to the baseline fEPSP slope values. The derived parameters (n = number of slices, N = number of animals) were compared using a one-way analysis of variance, which was followed by multiple comparisons (Tukey test) or an unpaired t-test with the level of significance set at *p* < 0.05. The quantified bands in the Western blots were also calculated and compared using an unpaired t-test with the level of significance set at *p* < 0.05. Also, the parameters and bars were expressed as the mean ± the standard error of the mean.

## Supplementary Information

Supplementary Information 1.

Supplementary Information 2.

## Data Availability

The datasets generated during and/or analyzed during the current study are available from the corresponding author upon reasonable request.

## Abbreviations

HCNP: Hippocampal cholinergic neurostimulating peptide
HCNP-pp: Hippocampal cholinergic neurostimulating peptide precursor protein
LTP: Long-term potentiation
fEPSPs: Field excitatory postsynaptic potentials
Ach: Acetylcholine
ChAT: Choline acetyltransferase
VAchT: Vesicular acetylcholine transporter
CHT1: High-affinity choline transporter
MSN: Medial septal nucleus
SO: Stratum oriens
SCs: Schaffer collateral-commissural fibers
SR: Stratum radiatum
CCh: Carbachol
Prz: Pirenzepine
NMDAR: N-Methyl-D-aspartic acid receptor
AMPAR: α-3-Hydroxy-5-methyl-4-isoxasole propionic acid receptor
