# Supplementary material for: Reduction of glutamatergic activity through cholinergic dysfunction in the hippocampus of hippocampal cholinergic neurostimulating peptide precursor protein knockout mice
Source: Sci Rep. 2022 Nov 10;12:19161. doi: 10.1038/s41598-022-23846-x (PMC9649636; doi:10.1038/s41598-022-23846-x)
Supplement: Supplementary file 1 — Supplementary Information 1. [file 41598_2022_23846_MOESM1_ESM.pptx]

## Slide 1
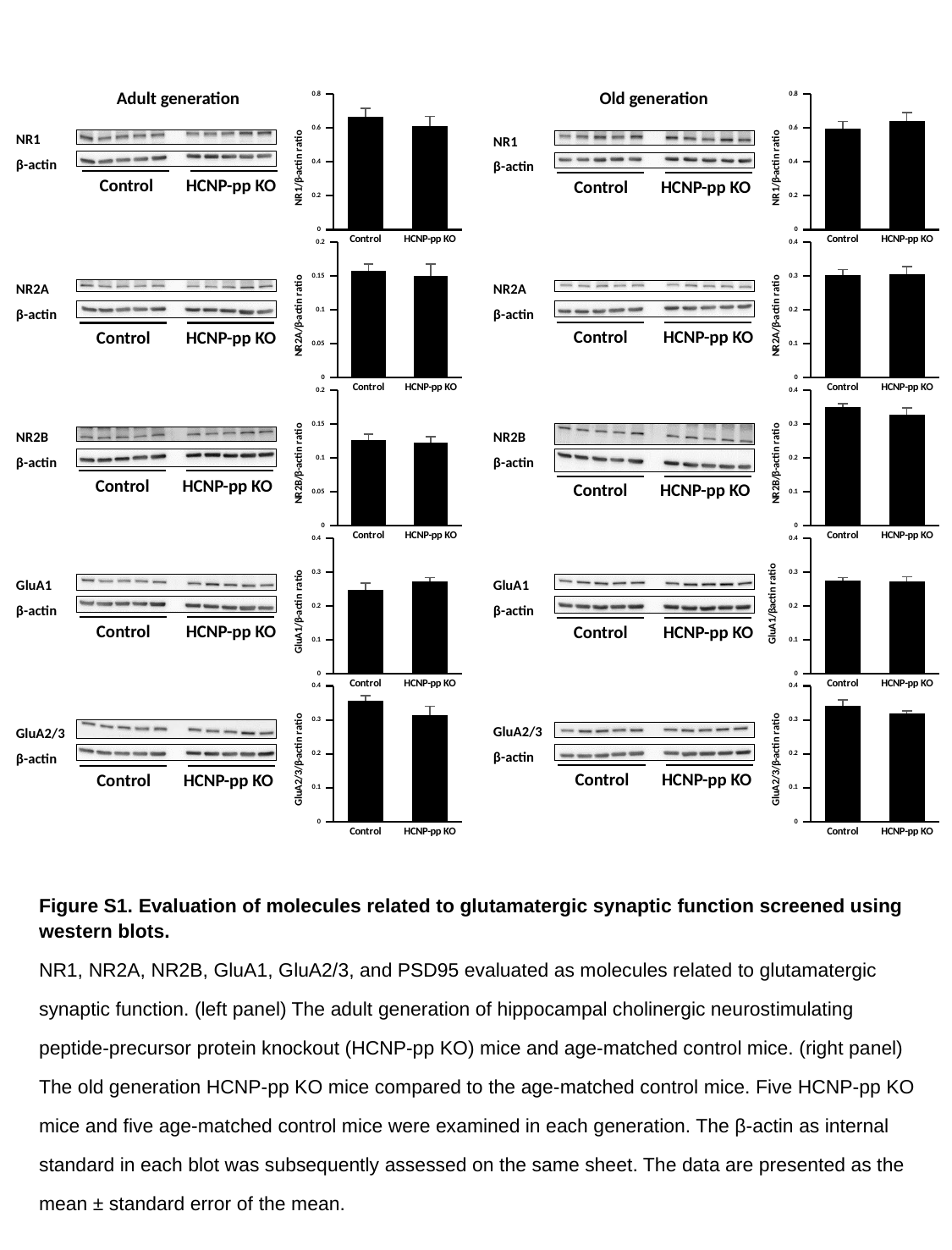

Adult generation
Old generation
### Chart
| Category | |
|---|---|
| Control | 0.6676756322708142 |
| HCNP-pp KO | 0.6102697351195361 |
### Chart
| Category | |
|---|---|
| Control | 0.5976851498501664 |
| HCNP-pp KO | 0.6431593990201143 |NR1
β-actin
NR1
β-actin
Control
HCNP-pp KO
Control
HCNP-pp KO
### Chart
| Category | |
|---|---|
| Control | 0.1579702403455934 |
| HCNP-pp KO | 0.15060629525106495 |
### Chart
| Category | |
|---|---|
| Control | 0.3038978580277417 |
| HCNP-pp KO | 0.30665952882556546 |NR2A
β-actin
NR2A
β-actin
Control
HCNP-pp KO
Control
HCNP-pp KO
### Chart
| Category | |
|---|---|
| Control | 0.12604899256542057 |
| HCNP-pp KO | 0.12282403945410529 |
### Chart
| Category | |
|---|---|
| Control | 0.34915612092553633 |
| HCNP-pp KO | 0.3286123534105294 |NR2B
β-actin
NR2B
β-actin
Control
HCNP-pp KO
Control
HCNP-pp KO
### Chart
| Category | |
|---|---|
| Control | 0.2480661187181254 |
| HCNP-pp KO | 0.271937608902754 |
### Chart
| Category | |
|---|---|
| Control | 0.27389755978164354 |
| HCNP-pp KO | 0.272367248922735 |GluA1
β-actin
GluA1
β-actin
Control
HCNP-pp KO
Control
HCNP-pp KO
### Chart
| Category | |
|---|---|
| Control | 0.3562015573811902 |
| HCNP-pp KO | 0.31467241294699544 |
### Chart
| Category | |
|---|---|
| Control | 0.34087044627909335 |
| HCNP-pp KO | 0.31787887390924 |GluA2/3
β-actin
GluA2/3
β-actin
Control
HCNP-pp KO
Control
HCNP-pp KO
Figure S1. Evaluation of molecules related to glutamatergic synaptic function screened using western blots.
NR1, NR2A, NR2B, GluA1, GluA2/3, and PSD95 evaluated as molecules related to glutamatergic
synaptic function. (left panel) The adult generation of hippocampal cholinergic neurostimulating
peptide-precursor protein knockout (HCNP-pp KO) mice and age-matched control mice. (right panel)
The old generation HCNP-pp KO mice compared to the age-matched control mice. Five HCNP-pp KO
mice and five age-matched control mice were examined in each generation. The β-actin as internal
standard in each blot was subsequently assessed on the same sheet. The data are presented as the
mean ± standard error of the mean.
