# Supplementary material for: Reduction of glutamatergic activity through cholinergic dysfunction in the hippocampus of hippocampal cholinergic neurostimulating peptide precursor protein knockout mice
Source: Sci Rep. 2022 Nov 10;12:19161. doi: 10.1038/s41598-022-23846-x (PMC9649636; doi:10.1038/s41598-022-23846-x)
Supplement: Supplementary file 2 — Supplementary Information 2. [file 41598_2022_23846_MOESM2_ESM.pptx]

## Slide 1
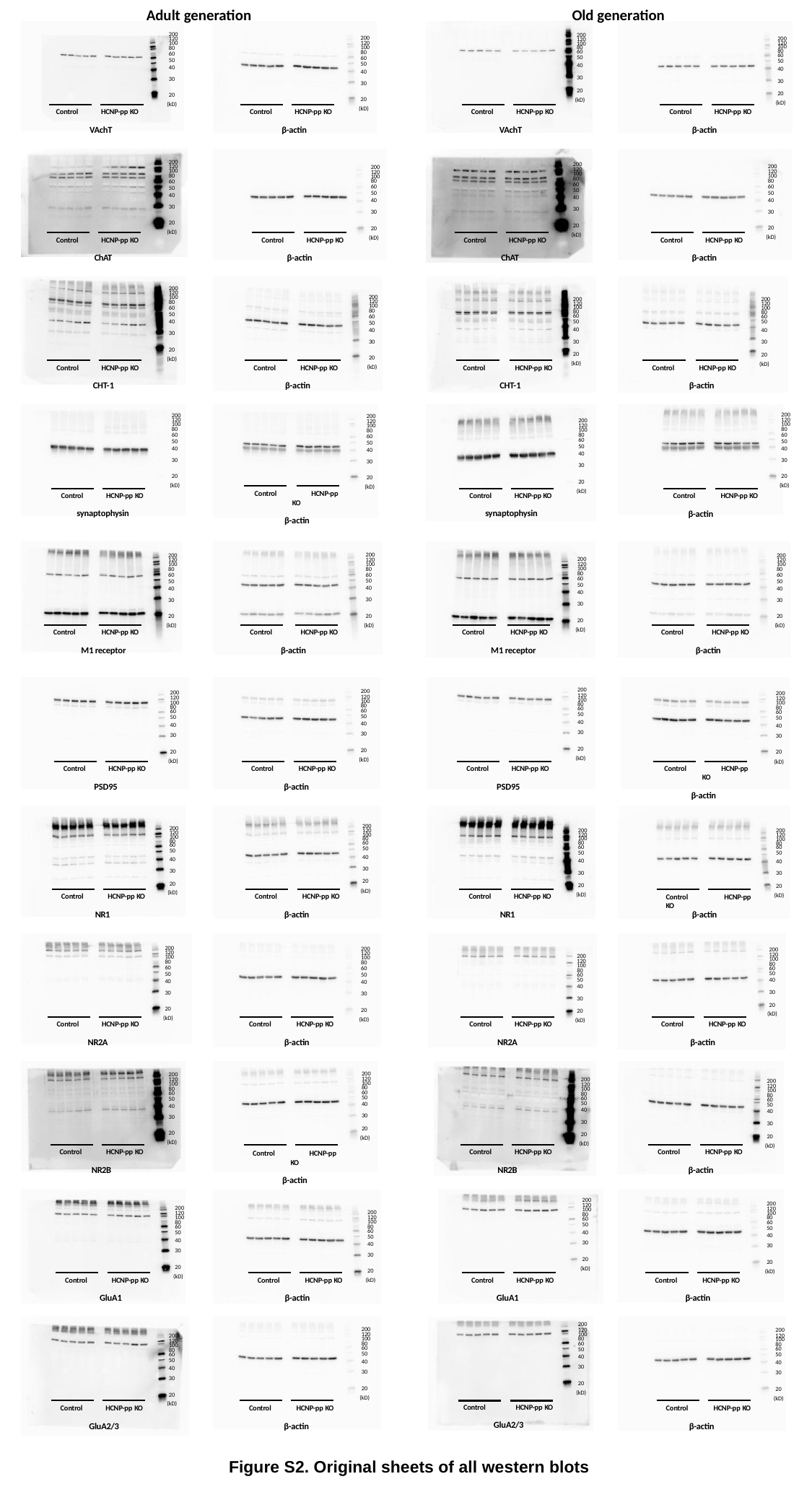

Adult generation
Old generation
200
120
100
80
60
50
40
30
20
(kD)
200
120
100
80
60
50
40
30
20
(kD)
200
120
100
80
60
50
40
30
20
(kD)
200
120
100
80
60
50
40
30
20
(kD)
Control
HCNP-pp KO
Control
HCNP-pp KO
Control
HCNP-pp KO
Control
HCNP-pp KO
VAchT
β-actin
VAchT
β-actin
200
120
100
80
60
50
40
30
20
(kD)
200
120
100
80
60
50
40
30
20
(kD)
200
120
100
80
60
50
40
30
20
(kD)
200
120
100
80
60
50
40
30
20
(kD)
Control
HCNP-pp KO
Control
HCNP-pp KO
Control
HCNP-pp KO
Control
HCNP-pp KO
ChAT
β-actin
ChAT
β-actin
200
120
100
80
60
50
40
30
20
(kD)
200
120
100
80
60
50
40
30
20
(kD)
200
120
100
80
60
50
40
30
20
(kD)
200
120
100
80
60
50
40
30
20
(kD)
Control	HCNP-pp KO
Control
HCNP-pp KO
Control
HCNP-pp KO
Control
HCNP-pp KO
CHT-1
β-actin
CHT-1
β-actin
200
120
100
80
60
50
40
30
20
(kD)
200
120
100
80
60
50
40
30
20
(kD)
200
120
100
80
60
50
40
30
20
(kD)
200
120
100
80
60
50
40
30
20
(kD)
Control	HCNP-pp KO
β-actin
Control	HCNP-pp KO
synaptophysin
Control	HCNP-pp KO
synaptophysin
Control
HCNP-pp KO
β-actin
200
120
100
80
60
50
40
30
20
(kD)
200
120
100
80
60
50
40
30
20
(kD)
200
120
100
80
60
50
40
30
20
(kD)
200
120
100
80
60
50
40
30
20
(kD)
Control
HCNP-pp KO
Control
HCNP-pp KO
Control
HCNP-pp KO
Control
HCNP-pp KO
M1 receptor
β-actin
M1 receptor
β-actin
200
120
100
80
60
50
40
30
20
(kD)
200
120
100
80
60
50
40
30
20
(kD)
200
120
100
80
60
50
40
30
20
(kD)
200
120
100
80
60
50
40
30
20
(kD)
Control	HCNP-pp KO
β-actin
Control
HCNP-pp KO
Control
HCNP-pp KO
Control
HCNP-pp KO
PSD95
β-actin
PSD95
200
120
100
80
60
50
40
30
20
(kD)
200
120
100
80
60
50
40
30
20
(kD)
200
120
100
80
60
50
40
30
20
(kD)
200
120
100
80
60
50
40
30
20
(kD)
Control	HCNP-pp KO
Control
HCNP-pp KO
Control
HCNP-pp KO
Control	HCNP-pp KO
NR1
β-actin
NR1
β-actin
200
120
100
80
60
50
40
30
20
(kD)
200
120
100
80
60
50
40
30
20
(kD)
200
120
100
80
60
50
40
30
20
(kD)
200
120
100
80
60
50
40
30
20
(kD)
Control
HCNP-pp KO
Control
HCNP-pp KO
Control
HCNP-pp KO
Control
HCNP-pp KO
NR2A
β-actin
NR2A
β-actin
200
120
100
80
60
50
40
30
20
(kD)
200
120
100
80
60
50
40
30
20
(kD)
200
120
100
80
60
50
40
30
20
(kD)
200
120
100
80
60
50
40
30
20
(kD)
Control	HCNP-pp KO
β-actin
Control	HCNP-pp KO
Control
HCNP-pp KO
Control
HCNP-pp KO
NR2B
NR2B
β-actin
200
120
100
80
60
50
40
30
20
(kD)
200
120
100
80
60
50
40
30
20
(kD)
200
120
100
80
60
50
40
30
20
(kD)
200
120
100
80
60
50
40
30
20
(kD)
Control	HCNP-pp KO
Control
HCNP-pp KO
Control
HCNP-pp KO
Control
HCNP-pp KO
GluA1
β-actin
GluA1
β-actin
200
120
100
80
60
50
40
30
20
(kD)
200
120
100
80
60
50
40
30
20
(kD)
200
120
100
80
60
50
40
30
20
(kD)
200
120
100
80
60
50
40
30
20
(kD)
Control	HCNP-pp KO
GluA2/3
Control
HCNP-pp KO
Control
HCNP-pp KO
Control
HCNP-pp KO
GluA2/3
β-actin
β-actin
Figure S2. Original sheets of all western blots
